# Stroke in Chagas disease: from pathophysiology to clinical practice

**DOI:** 10.1590/0037-8682-0575-2021

**Published:** 2022-06-06

**Authors:** Thaís Aparecida Reis Lage, Julia Teixeira Tupinambás, Lucas Bretas de Pádua, Matheus de Oliveira Ferreira, Amanda Cambraia Ferreira, Antonio Lucio Teixeira, Maria Carmo Pereira Nunes

**Affiliations:** 1 Universidade Federal de Minas Gerais, Programa de Pós-Graduação em Ciências da Saúde, Infectologia e Medicina Tropical, Belo Horizonte, Minas Gerais , Brasil.; 2 Universidade Federal de Minas Gerais, Faculdade de Medicina, Belo Horizonte, Minas Gerais, Brasil.; 3 Faculdade Ciências Médicas de Minas Gerais, Belo Horizonte, Minas Gerais, Brasil.; 4 University of Texas, Health Science Center at Houston, Neuropsychiatry Program, Houston, United States of America.

**Keywords:** Stroke, Chagas disease, Pathophysiology

## Abstract

Despite substantial progress toward its control, Chagas disease continues to be a major public health problem in Latin America and has become a global health concern. The disease affects approximately 6 million people, of whom 20-40% will develop cardiomyopathy over the years after the initial *Trypanosoma cruzi* infection. Chagas cardiomyopathy is the most serious and frequent manifestation of Chagas disease. Clinical manifestations vary widely according to the severity of myocardial dysfunction, ranging from asymptomatic to severe forms, including dilated cardiomyopathy with heart failure, arrhythmias, thromboembolism events, and sudden death. Chagas disease is a risk factor for stroke regardless of the severity of cardiomyopathy, which is a leading cause of chronic disability. Classically, stroke etiology in patients with Chagas disease is thought to be cardioembolic and related to apical aneurysm, mural thrombus, and atrial arrhythmias. Although most strokes are thromboembolic, other etiologies have been observed. Small vessel disease, atherosclerosis, and cryptogenic diseases have been reported in patients with Chagas disease and stroke. The potential mechanisms involved in non-embolic strokes include the presence of associated risk factors, pro-inflammatory and prothrombotic disease states, and endothelial dysfunction. However, the contribution of each mechanism to stroke in Chagas disease remains unclear. The review aims to provide an overview of stroke in Chagas disease, highlighting the main pathophysiological mechanisms, clinical presentation, approaches for prevention, and unanswered questions regarding treatment strategies.

## INTRODUCTION

Chagas disease, caused by the protozoan *Trypanosoma cruzi*, is the third most common cause of parasitic infection worldwide. Despite substantial progress toward its control, Chagas disease continues to be a major public health problem in Latin America and has become a global health concern. The disease affects approximately 6 million people of whom 20-40% will develop cardiomyopathy over the years after the initial *T. cruzi* infection^1^ ^-^ ^3^. Chagas cardiomyopathy is the most serious and frequent manifestation of Chagas disease. Clinical manifestations vary widely according to the severity of myocardial dysfunction, ranging from asymptomatic to severe forms, including dilated cardiomyopathy with heart failure, arrhythmias, thromboembolism events, and sudden death.

Chagas disease is a risk factor for stroke, independent of the severity of cardiomyopathy and is a leading cause of chronicdisability^4^. Classically, stroke etiology in patients with Chagas disease is thought to be cardioembolic owing to apical aneurysm, mural thrombus, and atrial arrhythmias^5^ ^,^ ^6^. Although the majority of strokes are thromboembolic, other etiologies, including small vessel disease, atherosclerosis, and cryptogenic, have been observed in Chagas disease^7^. The potential mechanisms involved in these non-embolic strokes include the presence of associated risk factors, pro-inflammatory and prothrombotic disease states, and endothelial dysfunction^5^. However, the contribution of each mechanism to stroke in Chagas disease remains unclear. The review aims to provide an overview of stroke in Chagas disease, highlighting the main pathophysiological mechanisms, clinical presentation, approaches for prevention, and unanswered questions regarding treatment strategies.

## CHAGAS DISEASE EPIDEMIOLOGY

Chagas disease remains a major cause of disability and cardiovascular death in Latin America. An estimated six million infected individuals live in South America, Central America, and Mexico. Over the last few decades, the global migration of individuals from highly endemic areas has taken Chagas disease to other countries, especially the United States. Vector-borne transmission is the main form of infection in endemic regions. Programs to control transmission, particularly in the southern cone countries of South America, have decreased the incidence of Chagas disease.

The impact of the disease can be noticed not only by the related mortality, which reaches values higher than several other contagious diseases such as schistosomiasis, leishmaniasis, and tuberculosis but also by the potential years of life lost with disabilities (DALYs)^8^. Much of this impact is due to secondary complications caused by the disease, including heart failure, and its complications were well intestinal involvement. 

## STROKE IN THE SETTING OF CHAGAS DISEASE: AN OVERVIEW OF PREVIOUS STUDIES

Several studies have evaluated the relationship between Chagas disease and stroke^4^ ^,^ ^6^ ^,^ ^7^ ^,^ ^9^ ^-^ ^14^. Cardioembolic events have been identified as the main cause of cerebrovascular events. Conversely, some studies have highlighted that patients with Chagas disease also have events of non-embolic etiology with other pathophysiological mechanisms involved^4^ ^-^ ^7^ ^,^ ^15^.

Autopsy studies, including patients with advanced cardiomyopathy, showed high rates of cerebral infarction in patients with Chagas disease with no previous clinical diagnosis^16^ ^,^ ^17^. A higher frequency of ischemic than hemorrhagic cerebrovascular events has been reported, with multiple foci of micronecrosis involving the cortical and subcortical regions. Cerebral infarcts were found in 10-35% of cases, as reported by a retrospective study autopsy^18^. The incidence of cardiac thrombosis in a series of 1,345 cases was 36% in patients with advanced heart failure and 15% in those with sudden death^19^. Cerebral atrophy, micronecrosis in the cerebral cortex, cortical laminar necrosis, and selective neuronal necrosis are other changes that can be observed in autopsy studies of patients with advanced Chagas heart disease^13^.

In contrast, epidemiological studies have reported a low incidence of thromboembolic events^9^ ^,^ ^11^ ^,^ ^20^. For example, Nunes et al. evaluating a cohort of 213 patients with Chagas disease and cardiomyopathy, showed an incidence rate of ischemic cerebrovascular events of 2.67 events per 100 patients/year^11^. The left ventricular ejection fraction and left atrial volume were independent risk factors for stroke^11^. In another study by Cerqueira-Silva et al., the incidence of stroke was 2.02 per 100 patients/year^21^.

A previous study showed that traditional vascular risk factors such as hypertension, diabetes mellitus, and smoking are less frequent in patients with stroke and Chagas disease than those without^22^. Stroke recurrence is estimated to occur in 20% of the patients^5^. Oliveira-Filho et al. evaluated 305 patients and demonstrated an independent association between Chagas disease and stroke, regardless of the severity of heart disease^6^, similar to other studies^12^ ^,^ ^23^ ^,^ ^24^. Jesus et. al. demonstrated an independent relationship between the signs of microembolism on transcranial Doppler and the history of stroke in patients with Chagas disease^25^. These data further corroborate the hypothesis that several risk factors are involved in addition to those already elucidated.

The cumulative risk of ischemic stroke and Chagas disease remains poorly understood because of the limited number of studies. A hospital-derived cohort of patients with mild-to-moderate heart failure demonstrated a low prevalence of stroke^9^. Although the studies are already being carried out, obtaining more robust data is limited by the lack of standardization in clinical assessment, neuroimaging examinations, and follow-up. Studies examining the risk factors for stroke in patients with Chagas disease are summarized in Table 1. 

**TABLE 1:** Studies on stroke in Chagas disease.

Author/year	Population included	Nº of patients with stroke	Characteristics of patients with Chagas disease	Main findings
Bestetti/2000^9^	79	1 fatal stroke	Mild to moderate heart failure	Prevalence of stroke is low in a hospital-derived cohort of patients
Aras/2003^17^	524 autopsies	92 with encephalic infarction	Patients who died from heart failure	Cerebral infarction was associated with death in 52% of the cases
Oliveira-Filho/2005^6^	305	32	Cardiomyopathy	Systolic dysfunction, presence of cardiac arrhythmias, cardioversion, and diabetes are predictors of stroke
Carod-Artal/2005^25^	478 with stroke	94 Chagas with stroke	Indeterminate form and heart disease	Apical aneurysm, heart failure, arrhythmia, female, and hypertension are predictors of stroke
Paixao/2009^14^	101 with stroke	101	Indeterminate form and heart disease	Previous stroke/transient ischemic attack history, atrial fibrillation, and CD-positive serology are associated with stroke
Nunes/2009^11^	213	39	Heart failure with LV systolic dysfunction	Left ventricular systolic dysfunction and left atrial volume enlargement are independent risk factors for stroke
Jesus/2011^24^	144 (62 with Chagas disease)	9	Heart failure	Chagas disease and stroke history are risk factors for microembolism
Dias Junior/2014^7^	52 with Chagas disease	26	Indeterminate form and heart disease	Apical aneurysms and intracavitary thrombi
Nunes/2015^4^	330	67	Chagas cardiomyoapthy	Apical aneurysm and left ventricular thrombus
Guedes/2016^42^	65	35	Indeterminate form, heart disease and cardiodigestive	Thromboembolic events, imbalanced. Expression of IL-10, FoxP3, and iNOS are associated with higher stroke and death risks
Montanaro/2016^45^	86 with Chagas and stroke	86	Patients with Chagas disease hospitalized with stroke	The FIOCRUZ score did not predict cardiomebolic stroke etiologies
Montanaro/2018^43^	279	279	Indeterminate form and heart disease	Age at stroke, initial modified Rankin Scale, bladder dysfunction, diabetes, and alcoholism are associated with mortality after stroke.
Cerqueira-Silva/2021^21^	565 (271 with Chagas disease)	16 with stroke during follow-up	Heart failure	Chagas disease is associated with increased risk of stroke and death independently of heart failure severity or cardiac arrhythmias
Montanaro/2021^44^	499	499	Patients with Chagas disease and stroke in several academic, hospital-based, and university hospitals across Brazil	Higher prevalence of vascular risk factors and lower median age in patients with cardioembolic etiology

## PATHOPHYSIOLOGICAL MECHANISM

Chagas cardiomyopathy (ChCM) is a highly embolic disease. Most strokes in patients with Chagas disease are related to mechanisms that predispose patients to thromboembolic events. The incidence of embolic events in these patients varies widely depending on the population included^20^. 

**FIGURE 1(A and B):**
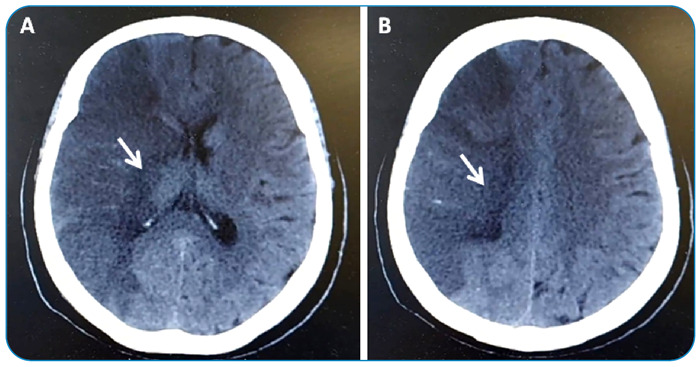
Ischemic stroke in the territory of the right middle cerebral artery (ischemic area indicated by white arrow) in a 54-year-old patient with Chagas disease. An echocardiogram showed reduced left ventricular ejection fraction with thrombus at apical aneurysm, classifying the stroke as cardioembolic.

Cardioembolism is the main mechanism of stroke in patients with Chagas disease (Figure 1)^7^. Several cardiac conditions have been proposed as sources of embolism in Chagas disease. The main reported risk factors for stroke are heart failure, apical aneurysm, left ventricular thrombus, severe atrial dilatation, left ventricular systolic dysfunction, advanced age, and atrial fibrillation^5^ ^,^ ^7^ ^,^ ^11^ ^,^ ^12^ ^,^ ^26^. However, stroke can occur in patients without clinical evidence of heart disease or other risk factors^5^ ^,^ ^12^. Additionally, stroke recurrence is estimated to occur in 20% of patients^5^.

**FIGURE 2:**
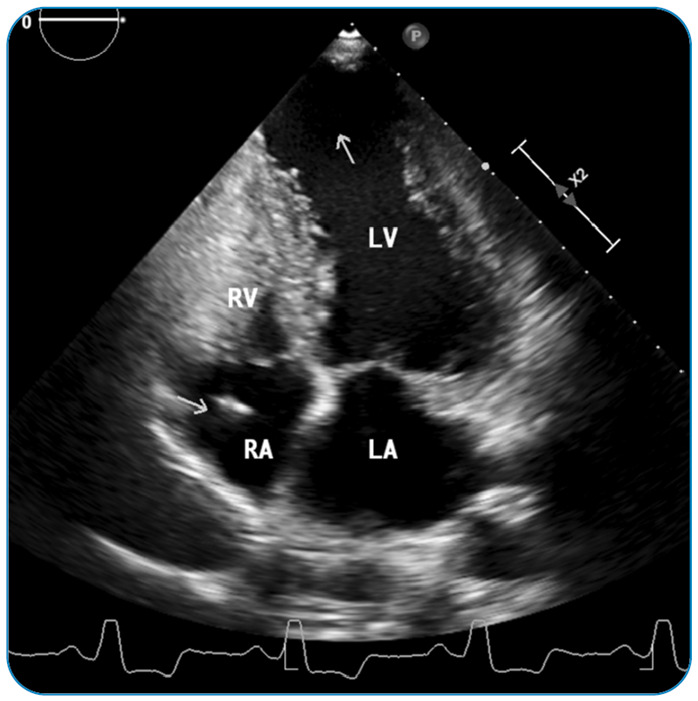
Echocardiographic image at the apical four-chamber view of a patient with Chagas disease presented with stroke. A large left ventricular apical aneurysm (white arrow) is seen. The right ventricle has a normal size with a pacemaker wire in the right atrium (arrow). **RV:** right ventricle, **LV:** left ventricle, **RA:** right atrium, **LA:** left atrium.

An apical aneurysm is a ventricular wall remodeling that can be seen at any stage of Chagas disease (Figure 2). The morphology of the aneurysm varies and may present as a small “hollow punch” or as a large aneurysm^27^ ^,^ ^28^. Left ventricular aneurysm predicts the development of mural thrombus and stroke^4^. Aneurysms^29^ can be found in diverse ventricle regions, including the apex, inferolateral wall, interventricular septum, and anterolateral walls^28^ ^,^ ^29^. Right ventricular aneurysms are uncommon; however, some patients have apical aneurysms that affect both ventricles. 

Atrial fibrillation is a well-established risk factor for stroke, regardless of the severity of left ventricular dysfunction severity^26^. A hospital-based case-control study showed that approximately 15% of patients with Chagas disease and stroke admitted to the hospital had atrial fibrillation^26^. Furthermore, a population-based cohort study showed that atrial fibrillation predicted the risk of stroke mortality in *T. cruzi* infected the older adults^30^. However, the risk of stroke varies among patients with atrial in relation to other clinical features. Left atrial enlargement is associated with stroke risk, which is independent of atrial ﬁbrillation, age, and other risk factors for cerebrovascular diseases^31^. 

Although cardioembolic strokes are the most common, atherothrombotic and small-vessel infarcts are also frequent etiologies of stroke in patients with Chagas disease^5^ ^,^ ^11^ ^,^ ^13^ ^-^ ^15^ ^,^ ^23^. Carod-Artal et al., including 136 patients with Chagas disease and stroke, showed that atherothrombotic and small vessels were the etiologies of stroke in 9% and 2% of patients, respectively^13^. The underlying mechanisms of these non-embolic strokes in Chagas disease seem to involve well-established risk factors for stroke in the general population (hypertension, hyperlipidemia, and smoking), pro-inflammatory milieu associated with prothrombotic disease, and the formation of atherosclerosis and endothelial dysfunction^10^ ^,^ ^26^ ^,^ ^32^. 

A cryptogenic stroke occurs with an increased frequency in patients with the indeterminate form of Chagas disease and mild heart disease. The etiology of ischemic stroke is cryptogenic in approximately 20-25% of patients with stroke and *T. cruzi* infection, and the etiology of ischemic stroke is cryptogenic^5^ ^,^ ^13^. However, the contributions of specific factors and mechanisms remain to be determined. The presence of a major cardiac source of embolism in the absence of arterial disease remains the mainstay for the clinical diagnosis of cardioembolic stroke. 

## CLINICAL PRESENTATION

Stroke in patients with Chagas disease presents clinically according to embolic events, which mainly affect the cortical zones and anterior circulation^7^ ^,^ ^26^. Clinical features that support the diagnosis of cardioembolic stroke include a sudden onset to the maximal deficit (< 5 min) and decreased level of consciousness at onset^33^. Up to 70% of patients present with immediate onset of partial anterior circulation syndrome, which includes two of the following three signs: motor or sensory deficit involving the face, arm, and leg; homonymous hemianopia; and higher cerebral dysfunction, such as aphasia or visual-field abnormalities^26^. Lacunar clinical presentations, lacunar infarcts, and especially multiple lacunar infarcts are unlikely^33^. 

**FIGURE 3:**
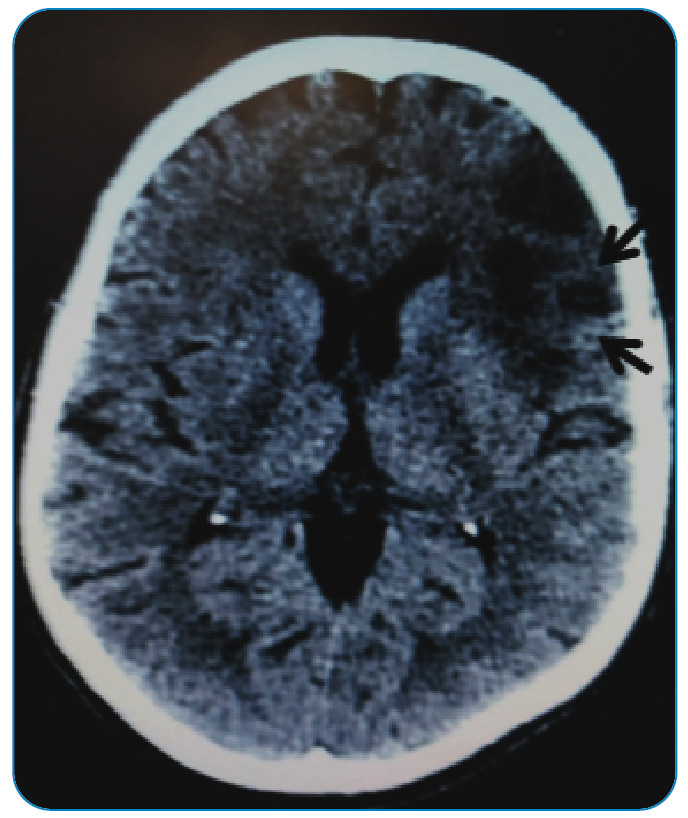
Stroke of atherosclerotic etiology in a 67-year-old patient with Chagas disease. The ischemic territory is indicated by a black arrow in the semioval center on the left. Significant intracranial atherosclerosis is detected.

In addition, patients with cardioembolism can present multiple events if secondary prophylaxis is not well established, presenting multiple deficits at distinct time points (Figure 3). Other clinical symptoms classically associated with cardioembolic infarction, such as headache and seizures at onset and onset during activity, are not specific to cardioembolic stroke^33^.

**FIGURE 4:**
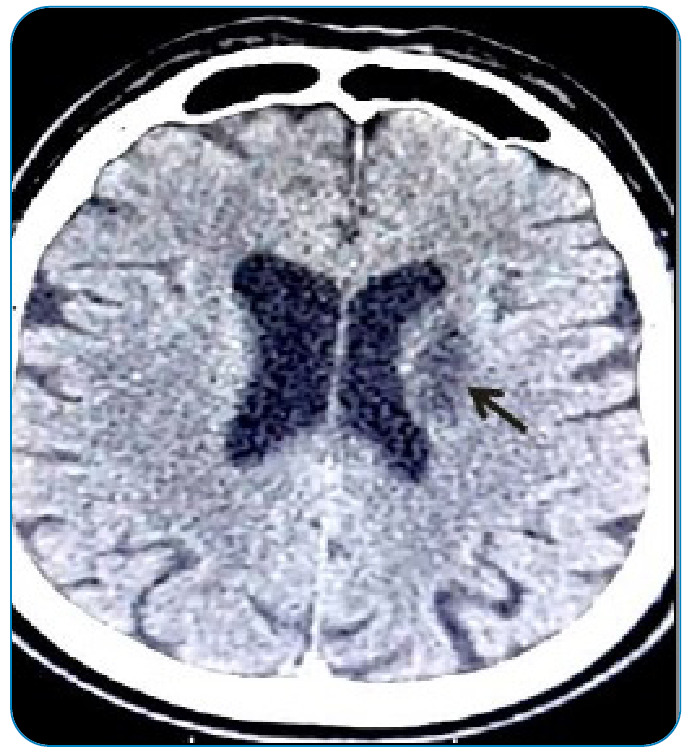
Patient with Chagas disease, 60 years old, with a history of multiple strokes. Areas of malacea were identified in the left frontal lobe, indicated by back arrows.

Ischemic stroke of atherosclerotic etiology has also been reported in patients with Chagas disease and is associated with classic cardiovascular risk factors. An aging population infected with *T. cruzi* is another additional risk that must be considered. An increased number of patients with Chagas disease and cardiovascular risk factors may increase the risk of stroke^5^ ^,^ ^10^. The clinical presentation is more related to lacunar events such as pure motor, sensorimotor, or pure sensory presentation, and the more restricted involvement seen in neuroimaging (Figure 4).

Some evidence suggests that *T. cruzi* damages cardiac muscle and vascular smooth muscle in acute infection in mice, causing generalized vasculitis; however, the chronic effect of acute vasculopathy in small vessels in the brain is still unknown^34^.

## STROKE TREATMENT

Current treatment for acute ischemic stroke in patients with Chagas disease follows the available guidelines for the general population^35^. Based on the 2019 stroke guideline^35^, every patient suspected of having a cerebrovascular event should be evaluated in the emergency department by a neurology team, neuroimaging should be performed in the emergency room, and the National Institutes of Health Stroke Scale (NIHSS) should be assessed to verify the severity of the event. Based on these results, the patient may be a candidate for thrombolysis, thrombectomy, or a conservative approach if there are contraindications for other treatment options. In the acute phase of stroke, there is no evidence to suggest that management should differ between patients with Chagas disease and other patients with ischemic stroke. Thrombolysis is safe in patients with Chagas disease without an increased risk of bleeding compared with other patients undergoing thrombolysis^36^ ^,^ ^37^.

Regarding secondary prevention, the classification of ischemic stroke is of paramount importance in defining the path to be followed. Patients presenting with cerebrovascular events with NIHSS less than or equal to three, that is, a minor stroke or high-risk transient ischemic attack (TIA) (ABCD2 score greater than or equal to 4), are eligible to double anti-aggregation for a short period of time^35^. Two randomized, multicenter, double-blind, controlled studies established the efficacy of short-term dual antiplatelet therapy. The Clopidogrel in High-risk patients with Acute Non-disabling Cerebrovascular Events (CHANCE) study evaluated 5,170 patients at high risk for non-disabling acute cerebrovascular events, minor stroke, or high risk using clopidogrel associated with aspirin^38^. The Platelet-Oriented Inhibition in New TIA and minor ischemic stroke (POINT) study evaluated a population similar to that in the CHANCE study. Compared with aspirin alone, aspirin plus clopidogrel resulted in fewer ischemic events but more severe bleeding^39^. However, patients with cardioembolic stroke, which cannot be generalized to patients with Chagas disease, were excluded from these studies.

In patients with a major stroke (NIHSS score >4), treatment with aspirin and statins is recommended, and the control of risk factors for atherosclerosis. After the acute phase, when the risk of bleeding is no longer significant, patients classified as cardioembolic must undergo anticoagulation therapy. However, in the acute phase, urgent anticoagulation is not recommended to prevent early recurrent stroke, interrupt neurological deterioration, or improve results after an event. Patients with Chagas disease had indications for anticoagulation in the presence of atrial fibrillation or left ventricular thrombus^40^. One of the most widely used anticoagulants is still warfarin due to its availability and lower cost. Another option is direct anticoagulants, which, despite their higher cost, have the convenience of fixed doses and do not require frequent laboratory control^40^ ^,^ ^41^. However, the use of direct anticoagulants for the prevention of stroke in patients with Chagas disease has not yet been randomized. Another point to be analyzed is when anticoagulation therapy should be started after the acute event. The risk of hemorrhagic transformation after a major ischemic stroke must be considered because injured tissue is more vulnerable to bleeding. In general, for ischemic stroke with large territories affected, the time to wait for anticoagulation is 14 days, moderate events approximately 7 days, and smaller events after 3 days^.^ However, this assessment must be performed on a case-by-case basis.

Secondary stroke prevention is extremely important, and acknowledging the different pathophysiological mechanisms is paramount. Chagas disease patients with significant cardiac alterations and the presence of events that point to cardioembolic etiology benefit from the use of anticoagulation. On the other hand, patients with events of atherosclerotic etiology might benefit from the use of anti-aggregation and statins for the stabilization of plaques^35^.

## PREVENTION STRATEGIES

Given the early mortality and severe disability caused by stroke in patients with Chagas disease, it is extremely important to consider strategies to prevent ischemic events in this population^40^. For primary prophylaxis, two important questions must be answered. Initially, cardioembolic risk stratification was evaluated, and the benefits of anticoagulation or antiplatelet drugs were weighted. It is also important to assess the bleeding risk and potential harm to therapy. As patients with Chagas disease usually have a low educational status and have more difficulties in medical follow-up, anticoagulation with warfarin may be challenging, and direct oral anticoagulants are not a reality in the public health system of Brazil. 

Patients with atrial fibrillation, permanent or paroxysmal, are a subgroup with well-established indications for anticoagulation. Left ventricular dysfunction, increased left atrial volume, apical aneurysm, intracardiac thrombi, and different types of cardiac arrhythmias are other risk factors for stroke in Chagas disease^40^. Although apical aneurysm is an independent risk factor for stroke, the indication for anticoagulation is controversial in the absence of a thrombus^4^.

Souza et al. published a study in 2007 evaluating the risk factors for stroke in Chagas disease and developed a scoring system to guide the decision of anticoagulation in this population^41^. A total of 1,043 patients were included and followed up for approximately 5.5 years, with a stroke incidence of 0.56% per year^41^. Four variables were included in the score, and their combination classified patients according to the annual incidence of stroke. In addition, systolic dysfunction (two points), apical aneurysm (one point), abnormal ventricular repolarization (one point), and age > 48 years (one point) were incorporated into the score. Anticoagulation should be initiated in patients with a score of 4 to 5 points; patients with a score ≤ of 1 have a low incidence of ischemic events, and aspirin or no treatment is suggested. For patients with a score of 2 or 3, the choice of treatment must be individualized, and the risks of bleeding and thromboembolic events must be weighed in this situation. Unfortunately, because of the small number of events, lack of external validation, and other study limitations, the general applicability of this scale is restricted to a few scenarios.

## CONCLUSIONS AND FUTURES PERSPECTIVES

In Chagas-associated stroke, an accurate definition of the mechanism of stroke is crucial to guide effective care and therapy. Previous studies have shed light on the complex interactions between Chagas disease and stroke. Further research is needed to provide an in-depth understanding of the risk of ischemic stroke in patients with Chagas disease. Strategies for improving risk factor control among patients with Chagas disease are fundamental for the primary prevention of stroke. Educational campaigns should be encouraged as a tool to prevent stroke and improve the management of the acute phase of stroke in Chagas disease.
